# Conservative management outcomes in acute pediatric mastoiditis

**DOI:** 10.1007/s00405-025-09523-5

**Published:** 2025-07-08

**Authors:** Jumana Diana Reda, Per Caye-Thomasen

**Affiliations:** 1 Department of Oto-Rhino-Laryngology, Head and Neck Surgery, and Audiology, Entrance 6, 3rd Floor, Section 6033, Copenhagen, Denmark; 2 Copenhagen Hearing and Balance Center, Copenhagen, Denmark; 3 Faculty of Health and Medical Sciences, University of Copenhagen, Copenhagen, Denmark; 4 Copenhagen University Hospital Rigshospitalet, Inge Lehmanns Vej 8, 2100 Copenhagen, Denmark

**Keywords:** Acute otitis media, Complications, Microbiology, Epidemiology

## Abstract

**Background:**

The aim of this study is to determine whether initial conservative surgical treatment of acute mastoiditis (AM) leads to an increase of morbidity, and to report on recent developments in epidemiology and microbiology.

**Methods:**

At the end of 2016, a new guideline for treatment of pediatric AM was introduced at our tertiary referral center. Mastoidectomy in case of a retroauricular abscess was changed to retroauricular needle aspiration or incision. All patients diagnosed with AM 2017—2021 were identified retrospectively in our patient file records and all relevant clinical data retrieved, including data on epidemiology and microbiology (*n* = 73; 26 with subperiosteal abscess).

**Results:**

Overall, the conservative management strategy led to resolved disease without further treatment in 94% of cases. Cases presenting with a subperiosteal abscess and receiving initial conservative surgical treatment healed in 92%, although 8% needed repeated aspiration or incision. Two children (8%) had secondary mastoidectomy due to disease progression. At 1.4%, complications were rare and the hospital stay short (mean 2.9 days). The incidence of AM was low during the Covid pandemic. Bacterial spectrum was comparable to earlier findings, resistance to penicillin/ampicillin slightly increased at 11%/33%.

**Conclusion:**

Conservative initial management of pediatric AM leads to favorable outcomes not inferior to a more aggressive approach including primary mastoidectomy in case of subperiosteal abscess. Complications are (as) rare and hospital stay shorter. Incidence of AM was lower during the Covid pandemic, but otherwise unchanged compared to previous years, despite intercurrent introduction of pneumococcal vaccines.

## Introduction

Acute otitis media (AOM) is a common pediatric infection, with a peak incidence from 6 months to 2 years [1]. AOM causes inflammation and fluid build-up in the middle ear, commonly spreading to the mastoid through the narrow passage from the middle ear, the aditus ad antrum. In most cases, the infection, inflammation and fluid clear with or without treatment. Occasionally however, the infection spreads to the mastoid air cells and thus lead to acute mastoiditis (AM) [2, 3], which may occur with or without a subperiosteal abscess.

Acute mastoiditis is considered a serious bacterial infection and is the most common intratemporal complication of AOM in the pediatric population [3]. Considering the proximity of the mastoid bone to the inner ear, the facial nerve and the central nervous system, and the potential development of further intra- or extracranial complications, early diagnosis and appropriate treatment is critical.

There has been a lack of consensus and explicit guidelines regarding the diagnosis and management of AM [4]. Traditionally, surgical mastoidectomy was the cornerstone of treatment. However, in recent years there has been a shift towards a more conservative approach for AM [5–8]. Mastoidectomy may thus be perceived as the secondary treatment option if initial treatment fails. Compared to mastoidectomy, conservative management includes everything from antibiotics alone to antibiotics in combination with simple surgical procedures, e.g. insertion of a tympanic membrane ventilation tube or/and retroauricular aspiration or incision.

As most AOM cases are self-limiting and due to low diagnostic accuracy and concern of spread of antibiotic resistance, Scandinavian and some other north-European countries have had a conservative approach to prescription of antibiotics for AOM for many years. Thus, these countries have established a consensus on refraining from initial use of antibiotics in the treatment of AOM in children above six months of age. Danish, Swedish, and Norwegian studies have showed that this restrictive use of antibiotics does not result in an increase in AM [9–11].

The aim of this study is to determine whether the combination of restrictive use of antibiotics in AOM combined with recently implemented conservative treatment guidelines for AM leads to an increase in the morbidity. Thus, we here report on the clinical outcomes of conservative management of AM over a period of 5 years in our tertiary referral center, including data on epidemiology and microbiology. The findings are compared to previously published data on a less conservative approach involving primary mastoidectomy in case of subperiosteal abscess, and discussed in the context of the Covid pandemic, as well as national implementation of pneumococcal vaccination.

## Methods

Beginning November 2016, a new clinical guideline for treatment of pediatric AM was introduced at our tertiary referral center. Recommendation of surgical mastoidectomy in case of retroauricular abscess was changed to a recommendation of retroauricular needle aspiration or incision (in combination with intravenous antibiotics and insertion of a tympanic membrane ventilation tube). In order to explore the consequences of this change of treatment recommendation, this paper describes the clinical findings and treatment outcomes during the five years followingly.

All patients diagnosed with AM between November 2016 and October 2021 were identified retrospectively in our patient file records. Our tertiary referral center covers East Denmark (Zealand and Bornholm; 2.7 mio inhabitants; 440.000 children below 15 years of age). Patients were identified using the International Classification of Diseases (ICD) codes H70.0 (acute mastoiditis) and H70.9 (mastoiditis, unspecified).

A total of 162 cases of AM were identified, of which 63 were excluded because of double registration. Eleven were excluded due to age > 15 years. Of the remaining 88 cases, 17 cases failed to meet the chosen diagnostic criteria for AM (Table 1). Thus, 71 cases of AM were included through this process.

**Table 1:** Criteria of acute mastoiditis. Modified from anthonsen et al. [11] Modified from anthonsen et al.

A) Clinical signs of recent (within 2 weeks) AOM or perioperative clinical findings of AOM
B) At least 3 of following 4 clinical finding
1) Protrusion of the pinna
2) Retroauricular redness
3) Retroauricular pain on palpation
4) Retroauricular swelling with or without fluctuation/subperiosteal abscess
C) And/or operative findings of acute mastoiditis
D) Exclusion of other conditions (e.g., external otitis, insect bite, erysipelas etc.)

To ensure that no cases with AM were missed, we also extracted data for children admitted with the diagnoses DH66.0 (acute suppurative otitis media), DH66.4 (suppurative otitis media, unspecified) and DH66.9 (otitis media, unspecified). Through this extraction, a total of 318 cases were identified. After excluding all cases aged above 15 years of age and all double registrations, a total of 138 cases remained. 138 medical records were reviewed for clinical findings in accordance with the diagnostic criteria for AM. Of the 138 cases, 16 children were already registered with AM. Among the remaining 122, two met the diagnostic criteria for AM.

As one patient had been admitted twice with AM during the 5-year period, a total of 73 cases in 72 patients were thus included in the study. Several variables were registered for all cases, including clinical and paraclinical findings, microbiology, treatment and complications.

Ethical approval was not necessary since no children were contacted in person. Approval for access to patient information was obtained by the Danish Patient Safety Authority (approval #31–1521-21.

## Results

### Demographics and epidemiology

Between November 2016 and October 2021, a total of 72 children (73 cases) were identified with AM, of which 33 (45%) were females and 40 (55%) males.

Patient age at admission ranged from 0–11 years, the average age being 1.8 years (median 1.3 years; range 0.1–11.7). At the time of admission, 59 (81%) children were younger than 2 years of age, while 14 (19%) were 2 years of age or older. Three children (4%) were 5 years or older. The average number of days admitted was 2.9 (median 2; range 1–13 days).

**Fig. 1:**
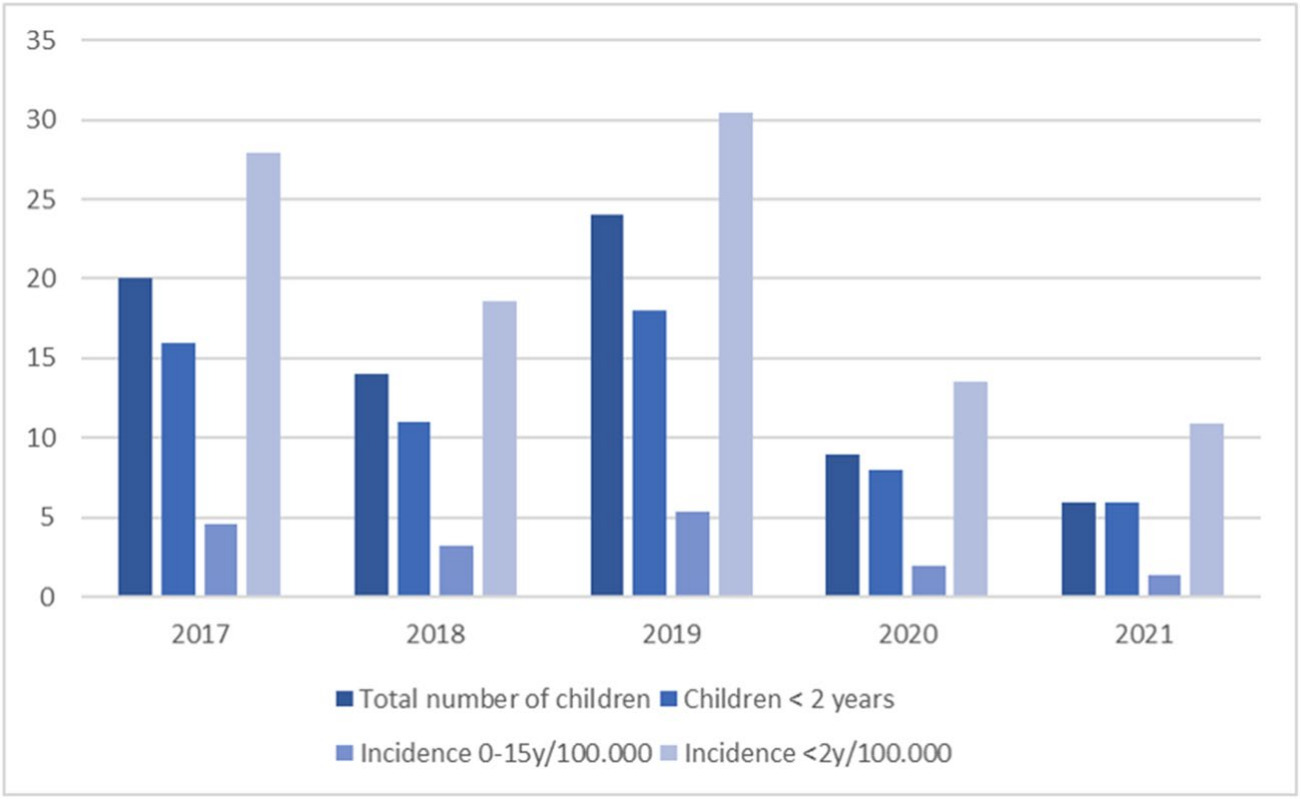
Incidence of acute pediatric mastoiditis in Eastern Denmark 2017–21 (2.7 mio inhabitants; 440.000 children)

The mean incidence of AM for the 5-year period was 3.3/100.000 children/year (range: 1.4–5.4). For children < 2 years of age, the mean incidence for the 5-year period was 20.3/100.000 children/year (range: 10.9–30.4). The incidence of AM varied over the 5-year period, as a lower incidence was found during the two latter years (2020 and 2021). Figure 1 illustrates the number of patients diagnosed per year and the fluctuation in incidence over the 5-year period.

### Clinical and paraclinical findings

The clinical findings are shown in Table 2. The occurrence of retroauricular abscess had been assessed by clinical finding (swelling and fluctuation), ultrasound examination and/or the extraction of pus by retroauricular needle aspiration or incision. An abscess was found in 26 cases (36%). The mean body temperature at admission was 38.4 ºC (median 38.4; range 36–40.2; *n* = 42). The mean blood leucocyte count was 14.6 billion/L (median 14.1; range 3.6–38.5, *n* = 64) and mean C-reactive protein serum level was 83.9 mg/L (median 70.5; range 2–319, *n* = 52).

**Table 2:** Clinical findings

Sign	*N* with data on finding	*N* with finding	Percent	Percent of all cases (73)
Protrusion of the pinna	68	67	99%	92%
Retroauricular redness	71	68	96%	93%
Retroauricular swelling	56	53	95%	73%
Fluctuation	54	18	33%	25%
Retroauricular pain on palpation	48	46	96%	63%
Retroauricular abscess	73	26	36%	36%

### Microbiology

**Fig. 2:**
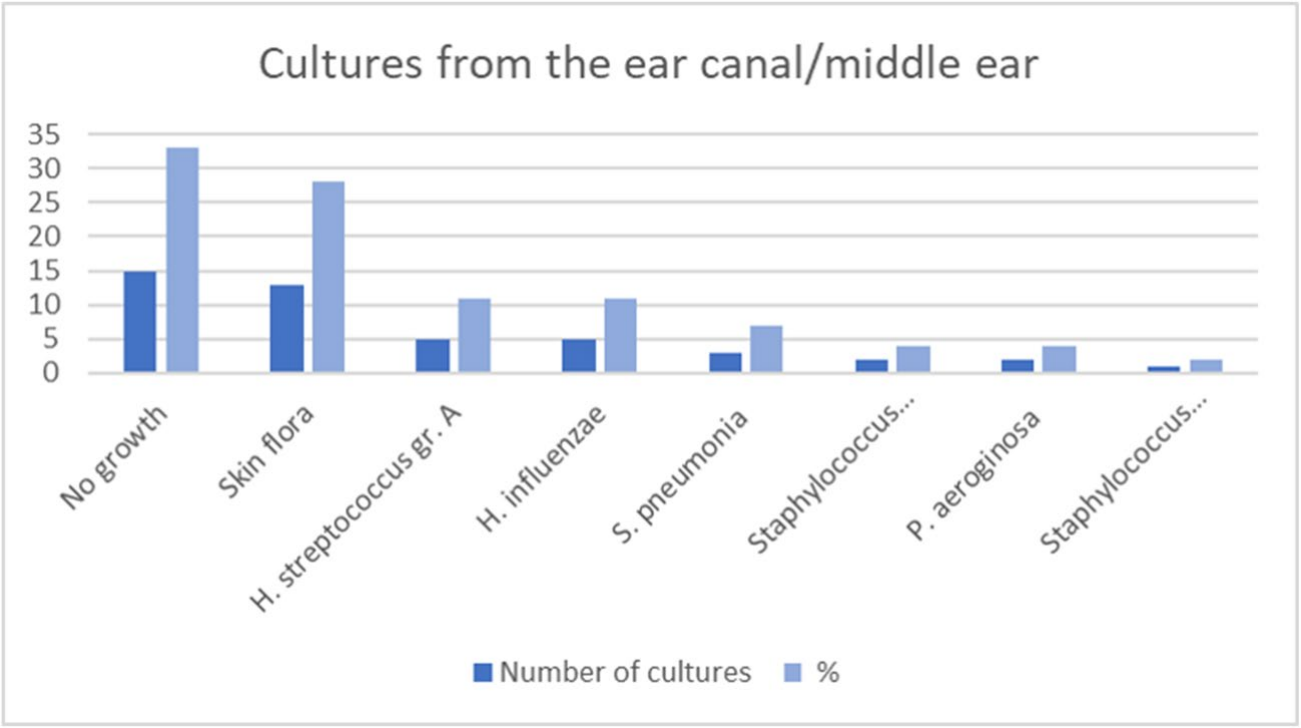
Microbiology, ear canal/middle ear (*n* = 46 of 73 cases). Susceptibility to penicillin/ampicillin was 61%/83%

Forty-seven (64%) of the 73 cases had cultures taken from the ear canal or the middle ear. One culture was lost, leaving 46 cultures for analysis. No bacterial growth occurred in 15 cases (33%), and skin flora in 13 cases (28%). A positive culture occurred in 18 cases (39%). The bacteria most commonly grown were *group A hemolytic Streptococcus* (11%) and *Haemophilus influenzae* (11%). The distribution of cultured bacteria is illustrated in Fig. 2. Of the 18 positive cultures, 61% were susceptible to penicillin, while 83% were susceptible to ampicillin.

**Fig. 3:**
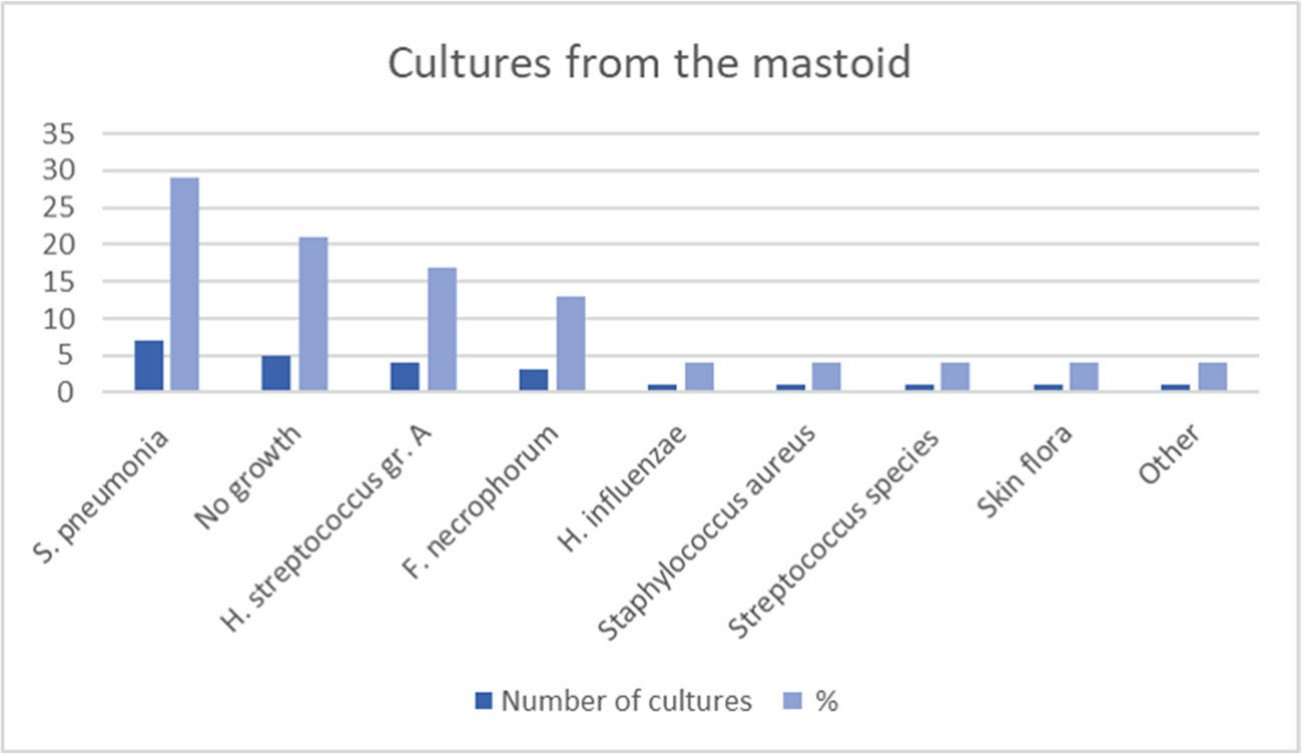
Microbiology, mastoid (*n* = 24 of 26 cases with subperiosteal abscess). The cultures were obtained by needle aspiration, incision, or mastoidectomy. Susceptibility to penicillin/ampicillin was 89%/67%

From the mastoid, all 26 cases with a retroauricular abscess had cultures taken, obtained by retroauricular needle aspiration or incision, in two cases at primary mastoidectomy (see “Surgical treatment” below). One culture was lost and another was never sent, leaving 24 cultures. The most common finding was *Streptococcus pneumoniae* (*n* = 7; 29%), the second most common finding was no growth (*n* = 5; 21%), followed by *group A hemolytic Streptococcus pneumoniae* (*n* = 4; 17%). The distribution of cultured bacteria is illustrated in Fig. 3. Of the 18 positive cultures from the mastoid (not including one with skin flora), susceptibility to penicillin was found in 16 cases (89%), whereas susceptibility to ampicillin occurred in 12 cases (67%). The two cultures from the mastoid that proved to be penicillin resistant showed S.aureus and H. influenzae.

### Antibiotic treatment

Prior to admittance, 62 cases (85%) did not receive antibiotics. Of the 11 cases (15%) who did, four (5%) had received penicillin, four (5%) amoxicillin and one (1%) received other antibiotics. Two cases did not have information available about the type of antibiotics administered.

Of the 11 cases (age 0.6–11.7 years) that were treated with antibiotics prior to admittance, four cases (36%) had been treated for one day, one (9%) for 4 days and two cases (18%) for 5 days. Four patients did not have available information about duration of treatment. Four of the 11 cases were below 2 years of age.

During admittance, all 73 cases (100%) were treated with antibiotics. Ten different antibiotics were used, and some combinations were seen. Sixty cases (82%) were administered a single antibiotic. Cefuroxime was the preferred drug (56%), followed by penicillin (23%). Two cases (3%) received amoxicillin.

Thirteen cases (18%) were switched from one to another antibiotic, in a few cases as a response to bacterial culture results. One case was changed because of initial clinical suspicion of meningitis, another due to development of sepsis and periorbital edema. One was changed from intravenous to oral antibiotics due to problems with the intravenous catheter. Most commonly, the change was from either penicillin to cefuroxime or vice versa (more rarely). Cases initially seen in the pediatric ward had been treated initially with amoxicillin, later changed to either penicillin or cefuroxime by ENT clinicians. As per our guidelines, all children were prescribed peroral amoxicillin for 7 days upon discharge.

### Surgical treatment

Of the 73 cases, 68 (93%) were treated surgically in some way (Table 3). 67 cases (92%) had a tympanic membrane ventilation tube inserted. The most common initial surgical treatment was insertion of ventilation tube in addition to either retroauricular needle aspiration or retroauricular incision, performed in respectively 60% and 21% of surgical treatment cases. 10% were treated with insertion of ventilation tube only. Mastoidectomy was performed in four patients (5%), two of which as part of the initial treatment (although not indicated according to our new guidelines).

**Table 3:** Initial surgical treatment

Procedure	*N* = 68	Percent	Percent of all cases (73)
Tympanic membrane ventilation tube only	7	10%	10%
Ventilation tube and retroauricular needle aspiration	44	65%	60%
Ventilation tube and retroauricular incision	15	22%	21%
Mastoidectomy	2	3%	3%

Twenty-six cases presented with a subperiosteal abscess upon admission. Ten of these (38%) were initially treated with a tube and retroauricular needle aspiration, 14 (54%) with a tube and retroauricular incision, whilst two (8%) had a mastoidectomy performed.

In three (30%) of the 10 subperiosteal abscess cases subjected to initial aspiration, this was insufficient, as the patients had to be retreated surgically. Of the 14 subperiosteal abscess cases subjected to initial incision only one (7%) required additional surgical treatment (*p* = 0.27; Fisher exact test).

### Treatment outcomes

The initial treatment strategy led to resolved disease in 69 of the 73 cases (95%). Mean duration of admission was 2.9 days (median 2 days; range 1–13 days). Although not included in our treatment guidelines, two cases with a subperiosteal abscess had a mastoidectomy performed as part of the initial treatment. Thus, 67 of 71 cases (94%) were treated successfully by initial conservative treatment, i.e. intravenous antibiotics, tympanic membrane ventilation tube, and aspiration or incision in case of subperiosteal abscess. Of these 71 cases, 24 (34%) had a subperiosteal abscess at admission.

In four cases (5%), the initial treatment was deemed inadequate during the first days after admission. They all had a subperiosteal abscess at admission.

Two of the four children had a secondary mastoidectomy (8% of the 24 abscess cases receiving initial conservative surgical treatment, i.e. aspiration or incision), one of them initially treated by ventilation tube and retroauricular needle aspiration, while the other was treated initially by a tube and retroauricular incision. The first child had a worsening in the condition the day after tube/aspiration, leading to a mastoidectomy. The second child had a recurrence of the abscess three days after tube/incision, leading to a mastoidectomy.

The third of the four children (4% of the 24 abscess cases receiving initial conservative surgical treatment) was initially treated by a tube and retroauricular needle aspiration and then retreated successfully with a retroauricular incision, as septicemia and periauricular/periorbital edema occurred during the days after initial treatment.

In the last of the four patients (4% of the 24 abscess cases receiving initial conservative surgical treatment), initial retroauricular needle aspiration was repeated successfully (a tube had also been inserted initially), as an abscess was found (again) later the same day.

All four patients were cured upon secondary treatment.

Thus, conservative surgical treatment measures (i.e. aspiration or incision in combination with a ventilation tube) were sufficient in 22 (92%) of 24 cases presenting initially with a subperiosteal abscess, although 2 of these needed repeated aspiration or incision (9%).

### Complications

The overall complication rate was 1.4%.

One child (1.4%) developed septicemia and periauricular, as well as periorbital edema during the days after initial retroauricular needle aspiration of a subperiosteal abscess (the case also described above, in the paragraph on treatment outcomes/inadequate treatment). A secondary retroauricular incision was performed and the child healed completely.

Three children (4.1%) had a lumbar puncture performed because of suspicion of meningitis, of which one child was found to have a parameningeal irritation. One child (1.4%) had a computed tomography (CT) scan performed, due to suspicion of intracranial complications, which was however not found.

No child was diagnosed with facial paresis, meningitis, sigmoid sinus thrombosis or intracranial abscess.

Recurrences of AM after two weeks is not interpreted or defined as a complication per se and is therefore not included in the overall complication rate. Two children (2.8%) had a recurrence of AM, one after 3 months and the other one after 2 years. Both presented with a subperiosteal abscess the first time, one having initial mastoidectomy (deviating from the conservative guideline), the other having initial incision.

## Discussion

### Clinical outcomes

Initial conservative treatment consisting of intravenous antibiotics, tympanic membrane ventilation tube, and aspiration or incision in case of subperiosteal abscess led to resolved disease in 94% of cases. The four initial treatment failures (5%) had all presented with initial subperiosteal abscess.

Of the 73 cases, 26 cases (36%) had a subperiosteal abscess at admission. Although our newly implemented treatment guidelines do not include mastoidectomy as initial treatment, two cases with subperiosteal abscess had had this procedure performed as part of the initial treatment. Thus, 24 cases with a subperiosteal abscess were initially treated conservatively, i.e. with intravenous antibiotics, tympanic membrane ventilation tube, and aspiration or simple incision. Of these, four (17%) had to be retreated surgically. Three of the four had had initial aspiration, whereas one had a simple, but apparently insufficient incision performed. All resolved after surgical re-treatment, which consisted of a mastoidectomy in two aspiration cases (8%), re-aspiration in one case (4%), and extended re-incision in the last case (4%). Thus, conservative surgical treatment measures (i.e. aspiration or incision in combination with a ventilation tube) were sufficient in 22 (92%) of 24 cases presenting initially with a subperiosteal abscess, although 2 of these needed repeated aspiration or incision (8%).

Aspiration had been performed as initial treatment in 10 (38%) of the 26 cases presenting with an abscess at admission. In three cases (30%), this was insufficient, as the patients had to be retreated surgically. Initial incision was performed in 14 (54%), of which only one (7%) required additional surgical treatment. Considering the limited number of cases and thus a potential type 2-error, the findings may indicate, that aspiration is an insufficient procedure in cases with subperiosteal abscess, although the number of treatment successes/failures is not statistically different from the incision procedure (*p* = 0.27; Fisher exact test).

Comparing the treatment outcomes with those of the previous and more aggressive surgical treatment strategy used in our department, i.e. initial mastoidectomy in all cases presenting with a subperiosteal abscess [9], we find no significant differences in treatment outcomes, need for secondary surgery (mastoidectomy), recurrences or complications (see below). The hospital stay was however shorter for the conservative strategy (mean 2.9 vs 4.7 days), which also the finding in a Swedish study [12].

### Recurrences and complications

No child was diagnosed with facial paresis, meningitis, sigmoid sinus thrombosis or intracranial abscess, but one child (1.4%) developed septicemia and periauricular, as well as periorbital edema during the days after initial retroauricular needle aspiration of a subperiosteal abscess (the case also described and discussed above). A retroauricular incision was performed and the child healed completely. Thus, the overall complication rate was 1.4%, compared to 1.9% occurring during our previous and more aggressive surgical treatment strategy [9].

Recurrences of mastoiditis after two weeks is not interpreted or defined as a complication per se and is therefore not included in the overall complication rate. Two children (2.8%) had a recurrence of acute mastoiditis, one after 3 months and the other one after 2 years. Both presented with a subperiosteal abscess the first time, one having initial mastoidectomy (deviating from the conservative guideline), the other having initial incision.

### Incidence of acute mastoiditis

Over the 5-year period 2017–21, we found a mean incidence of 3.3/100.000 children/year. For children less than 2 years of age, the incidence was 20.3/100.000 children/year (Fig. 1). Fluctuation between years was seen (range: 1.3–4.9/100.000 children/year), most noteworthy showing very low numbers during the Covid pandemic years 2020–21. Only nine children were diagnosed and treated in 2020, only six in 2021. This may very well be due to social distancing, hence a decrease in upper respiratory tract and middle ear infections, as well as complications thereof. The overall numbers are smaller than those reported for the 10-year 1998–2007 in the same uptake area, for which the overall incidence was 4.8/100.000 children/year [9]. This may again be explained by the very low numbers occurring during Covid, as the numbers for the first three years 2017–9 were equivalent to the findings during 1998–2007. Thus, an impact of the addition of the 7-valent pneumococcal vaccine to the national pediatric vaccination program in 2007 and the 13-valent in 2010 seems to be absent.

### Microbiology and antibiotics

Upon paracentesis, 64% of the 73 cases had cultures taken from the ear canal/middle ear, most frequently showing no growth or skin flora, followed by Group A hemolytic *streptococcus*, *Haemophilus influenzae* and *Streptococcus pneumoniae* (Fig. 2). Overall, the susceptibility to penicillin in cultures from the ear canal/middle ear was 61%, for ampicillin 83%.

Upon retroauricular aspiration, incision or mastoidectomy, all 26 children with presenting with a retroauricular abscess had cultures taken from the mastoid (two cultures were however lost), most frequently showing *Streptococcus pneumoniae*, no growth or Group A hemolytic *streptococcus* (Fig. 3). Susceptibility to penicillin in cultures from the mastoid was 89%, and to ampicillin 67%. The two cultures from the mastoid that proved to be penicillin resistant showed *S.aureus* and *H. influenzae.*

Comparing to previous findings in the same catchment area, the above bacterial spectrum is more or less the same, but with a somewhat changed resistance pattern, as 77%/94% of cultures from the ear canal/mastoid were susceptible to penicillin during the period 1998–2007 [9] vs 61%/89% during the period 2017–21, possibly indicating a slight change in pattern towards more widespread bacterial resistance.

This may at least in part be due to increased use of antibiotics with a broad spectrum, as the choice and use of antibiotics has changed over the years. Over half of all the current children received cefuroxime at admittance, whereas 23% had penicillin and 3% amoxicillin. Thus, the use of cefuroxime has increased by 36% since 2013, whereas penicillin use decreased by 6%. In addition, 18% had a change of the type of antibiotic used at least once. These patients were primarily seen in the pediatric ward and initially treated with amoxicillin, later changed to either penicillin or cefuroxime by ENT clinicians. The most common change was from penicillin to cefuroxime.

The diversity and changes of antibiotic choice may indicate a lack of knowledge update amongst attending general, pediatric and ENT physicians, underlining the need for continuous surveillance and dissemination of bacterial culture and resistance pattern results.

In Scandinavia intravenous G-benzyl penicillin is recommended as the first-choice drug for acute mastoiditis, only changing antibiotics according to culture resistance patterns if necessary, or in case of a prolonged or complicated clinical course. This approach still seems to be justified, as pneumococcal/streptococcal resistance to penicillin is still uncommon in the Scandinavian countries, arguably due to a generally restrictive policy of antibiotic administration, inter alia for AOM, and a preference of narrow spectrum or culture-based antibiotic choice.

Although an increasing body of literature indicates that initial conservative treatment of pediatric acute mastoiditis leads to outcomes not inferior to a more aggressive approach [5–8, 12], we cannot conclude whether the conservative approach is applicable at a more international or global level, since bacteriology and resistance patterns varies significantly worldwide [13, 14]. Furthermore, variable access to the health care system is also an issue to consider [15], recognizing that AM may display rapid progression and is a potentially deadly complication. In other words, a mastoidectomy may very well be the best initial treatment in cases with a subperiosteal abscess, depending on local bacterial virulence, resistance patterns and health care accessibility.

## Conclusion

Over the 5-year period 2017–21, we found that a more conservative initial management of acute pediatric mastoiditis leads to favorable outcomes not inferior to a more aggressive approach including primary mastoidectomy in case of subperiosteal abscess. Complications were (as) rare and hospital stay shorter. The incidence of AM was lower during the Covid pandemic, but otherwise unchanged compared to previous years, despite intercurrent addition of the 7- and later the 13-valent pneumococcal vaccine to the vaccination program. The bacterial spectrum was comparable to earlier findings, although resistance to penicillin/ampicillin was slightly increased.
